# Development and validation of a nomogram based on biparametric MRI PI-RADS v2.1 and clinical parameters to avoid unnecessary prostate biopsies

**DOI:** 10.1186/s12880-023-01074-7

**Published:** 2023-08-15

**Authors:** Yunhan Wang, Lei Wang, Xiaohua Tang, Yong Zhang, Na Zhang, Biao Zhi, Xiangke Niu

**Affiliations:** 1 Department of Urology, Affiliated Hospital of Chengdu University, Chengdu, 610081 Sichuan China; 2 Department of Radiology, Ninety-Three Hospital, Jiangyou City, 610000 Sichuan China; 3 Department of Radiology, DeYang People’s Hospital, Deyang City, 610000 Sichuan China; 4 Department of General Practice Medicine, Affiliated Hospital of Chengdu University, Chengdu, 610081 Sichuan China; 5 Department of Interventional Radiology, Affiliated Hospital of Chengdu University, Chengdu, 610081 Sichuan China; 6 Department of Interventional Radiology, School of Medicine, Sichuan Cancer Hospital & Research Institute, University of Electronic Science and Technology of China (UESTC), Chengdu, 610041 China

**Keywords:** Biopsy, Magnetic resonance imaging, Multivariable risk stratification, Prostate cancer, Prostate Imaging Reporting and Data System

## Abstract

**Background:**

Biparametric MRI (bpMRI) is a faster, contrast-free, and less expensive MRI protocol that facilitates the detection of prostate cancer. The aim of this study is to determine whether a biparametric MRI PI-RADS v2.1 score-based model could reduce unnecessary biopsies in patients with suspected prostate cancer (PCa).

**Methods:**

The patients who underwent MRI-guided biopsies and systematic biopsies between January 2020 and January 2022 were retrospectively analyzed. The development cohort used to derive the prediction model consisted of 275 patients. Two validation cohorts included 201 patients and 181 patients from 2 independent institutions. Predictive models based on the bpMRI PI-RADS v2.1 score (bpMRI score) and clinical parameters were used to detect clinically significant prostate cancer (csPCa) and compared by analyzing the area under the curve (AUC) and decision curves. Spearman correlation analysis was utilized to determine the relationship between International Society of Urological Pathology (ISUP) grade and clinical parameters/bpMRI score.

**Results:**

Logistic regression models were constructed using data from the development cohort to generate nomograms. By applying the models to the all cohorts, the AUC for csPCa was significantly higher for the bpMRI PI-RADS v2.1 score-based model than for the clinical model in both cohorts (*p* < 0.001). Considering the test trade-offs, urologists would agree to perform 10 fewer bpMRIs to avoid one unnecessary biopsy, with a risk threshold of 10–20% in practice. Correlation analysis showed a strong correlation between the bpMRI score and ISUP grade.

**Conclusion:**

A predictive model based on the bpMRI score and clinical parameters significantly improved csPCa risk stratification, and the bpMRI score can be used to determine the aggressiveness of PCa prior to biopsy.

**Supplementary Information:**

The online version contains supplementary material available at 10.1186/s12880-023-01074-7.

## Background

Prostate cancer (PCa) is the second most common cancer in males worldwide [1]. Standard screening using prostate-specific antigen (PSA) levels has led to an overall reduction in mortality from PCa. Regrettably, PSA screening is also associated with the underdetection of clinically significant prostate cancer (csPCa) and the overdetection of clinically insignificant cancer [2]. Clinically insignificant cancers are cancers that are unlikely to progress or affect a man's life expectancy and therefore do not require immediate treatment. In contrast, csPCa exhibits greater aggressiveness and has a higher mortality rate. Prostate biopsy is the traditional diagnostic pathway in the detection of csPCa. Despite positive improvements in prebiopsy preparation, hematuria, hematospermia and infections remain the main adverse effects after the procedure. A noninvasive, easy-to-administer testing pathway that accurately diagnoses csPCa and ultimately avoids unnecessary biopsies is a major unmet need.

Risk-based patient selection for prostate biopsy has been used in daily clinical practice, either through empirical judgment or through the use of risk calculators. The use of multivariate risk calculators, such as the European Randomized Study of Screening for Prostate Cancer (ERSPC) and Prostate Cancer Prevention Trial Risk Calculator (PCPT-RC), has been demonstrated to avoid 20–35% of unnecessary biopsies in a number of external validation studies [3, 4]. Multiparametric magnetic resonance imaging (mpMRI) of the prostate has significantly improved the diagnostic accuracy for csPCa. The Prostate Imaging and Reporting Data System (PI-RADS) was first developed to standardize prostate MRI acquisition, data reporting, and interpretation, with version 1 published in 2012 and version 2.1 published in 2019 [5]. However, up to 24% of patients with a Gleason grade ≥ 2 may still be missed by mpMRI alone [2, 6]. Recent studies have shown that mpMRI can improve csPCa detection and risk stratification when incorporated into the available clinical parameters [7–9]. Unfortunately, the full PI-RADS compliant protocol is time-consuming (~ 40 min), and expensive testing might be difficult to implement on a large scale, especially for active surveillance. It is noteworthy that the cohorts reported by Zhang et al. [10] and Tamada et al. [11] demonstrated that the diagnostic performance of biparametric MRI (bpMRI) was comparable to that of mpMRI by using PI-RADS v2.1 to detect csPCa. Theoretically, a predictive model based on this faster, cheaper and contrast-free bpMRI protocol may be an effective way to safely reduce unnecessary prostate biopsies.

Early diagnosis and timely treatment of csPCa can help improve the life expectancy of patients with PCa [12]. Although there are many different treatment options for PCa, aggressive PCa requires urgent, curative treatment [13]. However, active surveillance is a possible strategy for clinically insignificant PCa. Since mpMRI is part of the diagnostic pathway, it is necessary to use simple MRI-derived parameters to assess the aggressiveness of PCa. Previous studies have shown that quantitative MRI parameters can be used to assess tumor aggressiveness [14, 15]. However, due to inconsistent methods in different studies, heterogeneous results have been observed.

The objectives of this study were (1) to investigate the added value of the bpMRI PI-RADS v2.1 score for a clinical-only model to avoid unnecessary prostate biopsies and (2) to demonstrate whether there is a correlation between the simple bpMRI-derived score and PCa aggressiveness.

## Methods

### Study design and participants

A total of 815 consecutive men with clinically suspected PCa who underwent mpMRI and subsequent targeted biopsy (TBx) combined with systemic biopsy (SBx) between January 2020 and January 2022 were included in the institutional review board-approved databases of the development cohort (*n* = 345), the validation cohort 1(*n* =245), and the validation cohort 2 (*n* = 225). Clinical characteristics (i.e., PSA level, age, digital rectal examination (DRE) findings) at the time of enrollment were obtained. Eligible participants included males aged > 50 years with clinical suspicion of PCa due to elevated PSA levels (> 4 ng/ml) or suspicious DRE exams who were recommended for an initial biopsy. Men with a history of treatment for benign/malignant prostate disease within the last 3 months were excluded. Men with clinical signs of urinary tract infection (including prostatitis) and inadequate image quality were also excluded. The research ethics committees of all institutions approved the retrospective study and all methods were carried out in accordance with relevant guidelines and regulations.

### Sample size and sensitivity analysis

There is no accepted method to estimate the sample size required to develop a predictive model; however, an established rule of thumb is to ensure that at least 10 events per predictive parameter are considered for inclusion in the predictive model equation [16]. Based on previous studies, the prevalence of csPCa exceeds 35% [2, 17, 18]; therefore, we set the event fraction at 34%, with 8 candidate predictors and a mean absolute prediction error (MPSE) of 0.09 for this study. We ultimately needed at least 240 patients to meet the requirements, and the calculation tool is available online (https://mvansmeden.shinyapps.io/BeyondEPV/). We conducted a sensitivity analysis to assess the net benefit (NB) of excluding missing values in the validation cohorts.

### bpMRI protocol

The bpMRI imaging acquisition protocol was in compliance with the PI-RADS v2.1 criteria, which includes high-resolution axial and sagittal T2-weighted imaging (T2WI), and axial diffusion-weighted imaging (DWI) [5]. Details of the bpMRI scan parameters are provided in the supplemental materials (Table S 1).

### Prostate biopsy

Patients at all 3 institutions underwent MRI-guided cognitive targeted biopsies and subsequent SBx. MRI-guided cognitive targeted biopsies were performed for each lesion with a PI-RADS v2.1 score ≥ 3. A minimum of 2 cores from each MRI target is recommended, with a greater number of cores at the operator's discretion based on lesion size and location and confidence in targeting accuracy. After performing the MRI-guided biopsies, a 12-core SBx was performed. Details of the biopsy procedure have already been reported in previous studies [19, 20]. Gleason scores were obtained according to the 2014 International Society of Urological Pathology (ISUP) consensus guidelines [21] by experienced pathologists at each institution (Table S 2). csPCa was defined as an ISUP grade ≥ 2.

### Image analysis

In each institution, bpMR images were presented to a single experienced genitourinary radiologist (Table S 2) in random order. The radiologists were blinded to the clinical information of the patients. Each reader retrospectively reviewed the images independently and determined the bpMRI score by applying the PI-RADS v2.1 criteria [5]. If more than one lesion was present, the lesion with the highest PI-RADS v2.1 score was used as the index lesion. Prostate volume (PV) was determined with T2-weighted images, defined as π/6 × length × width × height. Prostate-specific antigen density (PSAd) was equal to the total serum PSA level divided by PV.

### Statistical analysis

Model development, validation, and reporting were performed according to the Transparent Reporting of Multivariate Predictive Models for Individual Prognosis or Diagnosis (TRIPOD) guidelines [22]. We used Wilcoxon rank sum tests (continuous variables) or chi-squared analysis (categorical variables) to assess the baseline characteristics of the patients. Univariate and multivariate logistic regression analyses were conducted to generate two nomograms based on clinical parameters (clinical model) and the bpMRI PI-RADS v2.1-based model. The receiver operating characteristic (ROC) curves of the logistic regression models were used to assess the performance of each model, and the DeLong test was utilized to determine significant differences between AUCs. The calibration was explored graphically by constructing calibration plots.

Decision curve analysis (DCA) was carried out to evaluate and compare the clinical utility of the constructed models. To use DCA appropriately, we first determined whether the model outperformed another within a reasonable risk threshold range (10–20%). Second, we considered whether there were important additional costs (testing trade-offs) of using the model [23]. Net reduction curves were used to estimate the overall number of biopsies that could be avoided without missing any cancers when the number of patients was standardized to 100. Spearman correlation coefficients were calculated between ISUP grade and the clinical parameters as well as the bpMRI PI-RADS v2.1 score in the total included study population (*n* = 657). The strength of the correlation was classified as small (< 0.3), medium (0.3–0.5) or large (> 0.5). Statistical significance was set at *p* < 0.05. Statistical analyses were performed using R software (version 3.3.1; R Foundation for Statistical Computing, Vienna, Austria). The representative R codes used in this study are presented in the Supplementary table S 3.

## Results

### Patient characteristics and bpMRI results

**Fig. 1:**
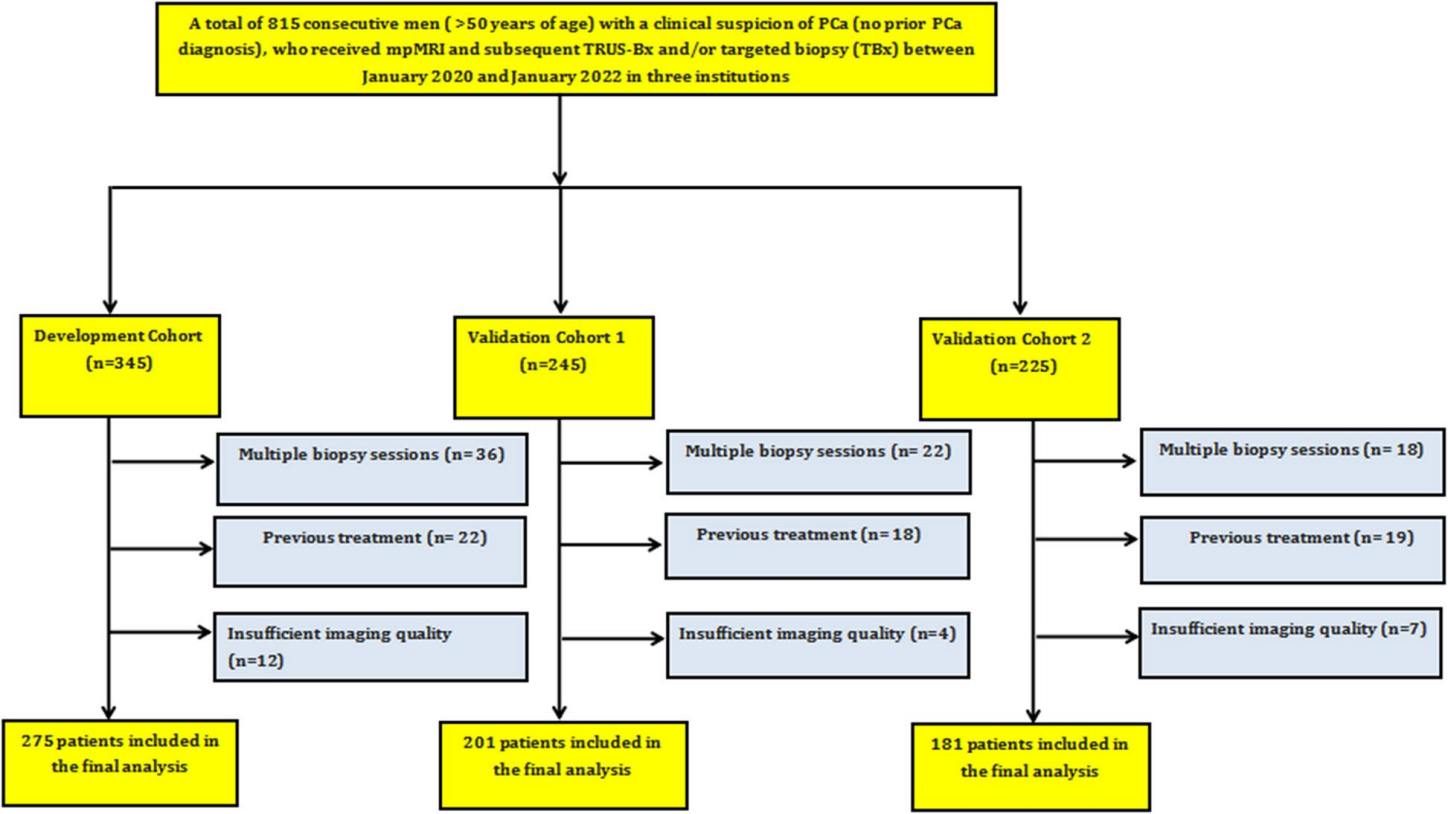
Flowchart showing the patient selection process

A total of 657 men were retrospectively included; 158 men were excluded for various reasons (Fig. 1). The development cohort, validation cohort 1 and validation cohort 2 consisted of 275, 201, and 181 patients, respectively. Table 1 summarizes the patient demographics for all cohorts. The prevalence of csPCa was 35% (*n* = 97) in the development cohort (median age at biopsy, 73 years), 38% (*n* = 77) in validation cohort 1 (median age at biopsy, 71 years), and 39% (*n* = 73) in validation cohort 2 (median age at biopsy, 74 years).

**Table 1:** Patient demographics of the development and validation cohorts

Variable	Development Cohort: Institution 1 (*n* = 275)	Validation cohort		*p* Value
		**Institution 2 (*n* = 201)**	**Institution 3 (*n* = 181)**	
**Median age at biopsy, yrs (IQR)**	73 (66, 78)	71 (67, 76)	74 (69, 78)	0.130^*^
**Family history of prostate cancer, No. (%)**				**0.500**^**×**^
**Positive**	115 (42%)	94 (47%)	83 (46%)	
**Negative**	129 (47%)	80 (40%)	80 (44%)	
**Unknown**	31 (11%)	27 (13%)	18 (9.9%)	
**DRE findings, No. (%)**				** > 0.900**^**×**^
**Normal**	127 (46%)	93 (46%)	90 (50%)	
**Abnormal**	121 (44%)	86 (43%)	76 (42%)	
**Unknown**	27 (9.8%)	21 (10%)	15 (8.3%)	
**Median PSA, ng/ml (IQR)**	13 (7, 24)	12 (8, 19)	13 (8, 22)	0.700^*^
**Median prostate volume, ml (IQR)**	50 (33, 71)	57 (40, 80)	58 (42, 78)	0.014^*^
**Median PSAd, ng/ml/ml (IQR)**	0.25 (0.13, 0.49)	0.19 (0.11, 0.41)	0.20 (0.11, 0.44)	0.031^*^
**PI-RADS v2.1 category, No. (%)**				**0.058**^**×**^
1	58 (21%)	24 (12%)	24 (13%)	
2	43 (16%)	38 (19%)	42 (23%)	
3	53 (19%)	56 (28%)	47 (26%)	
4	62 (23%)	33 (16%)	27 (15%)	
5	59 (21%)	50 (25%)	41 (23%)	
**Pathology results**				**0.078**^**×**^
No PCa	142 (51%)	98 (49%)	87 (48%)	
ISUP grade 1	36 (13%)	26 (13%)	21 (12%)	
ISUP grade 2	53 (19%)	36 (18%)	26 (14%)	
ISUP grade 3	19 (7%)	15 (7%)	22 (12%)	
ISUP grade 4	16 (6%)	17 (9%)	12 (6%)	
ISUP grade 5	9 (3%)	9 (4%)	13 (7%)	

^*^ Represents Kruskal‒Wallis rank sum test; × represents Pearson's Chi-squared test; *IQR* interquartile range, *No* number, *DRE* digital rectal examination, *IQR* interquartile range, *PSA* prostate-specific antigen, *PSAd* prostate-specific antigen density, *PI-RADS v2.1* Prostate Imaging Reporting and Data System version 2.1, *PCa* prostate cancer, *ISUP* International Society of Urological Pathology

The development cohort had a similar age, family history, DRE results, and median PSA level compared to the validation cohort but had a significantly lower median prostate volume, a higher proportion of median PSAd values. Based on bpMRI results and pathology findings, there was no significant difference between the three groups.

### Development of the predictive model and ROC curve analysis

**Fig. 2:**
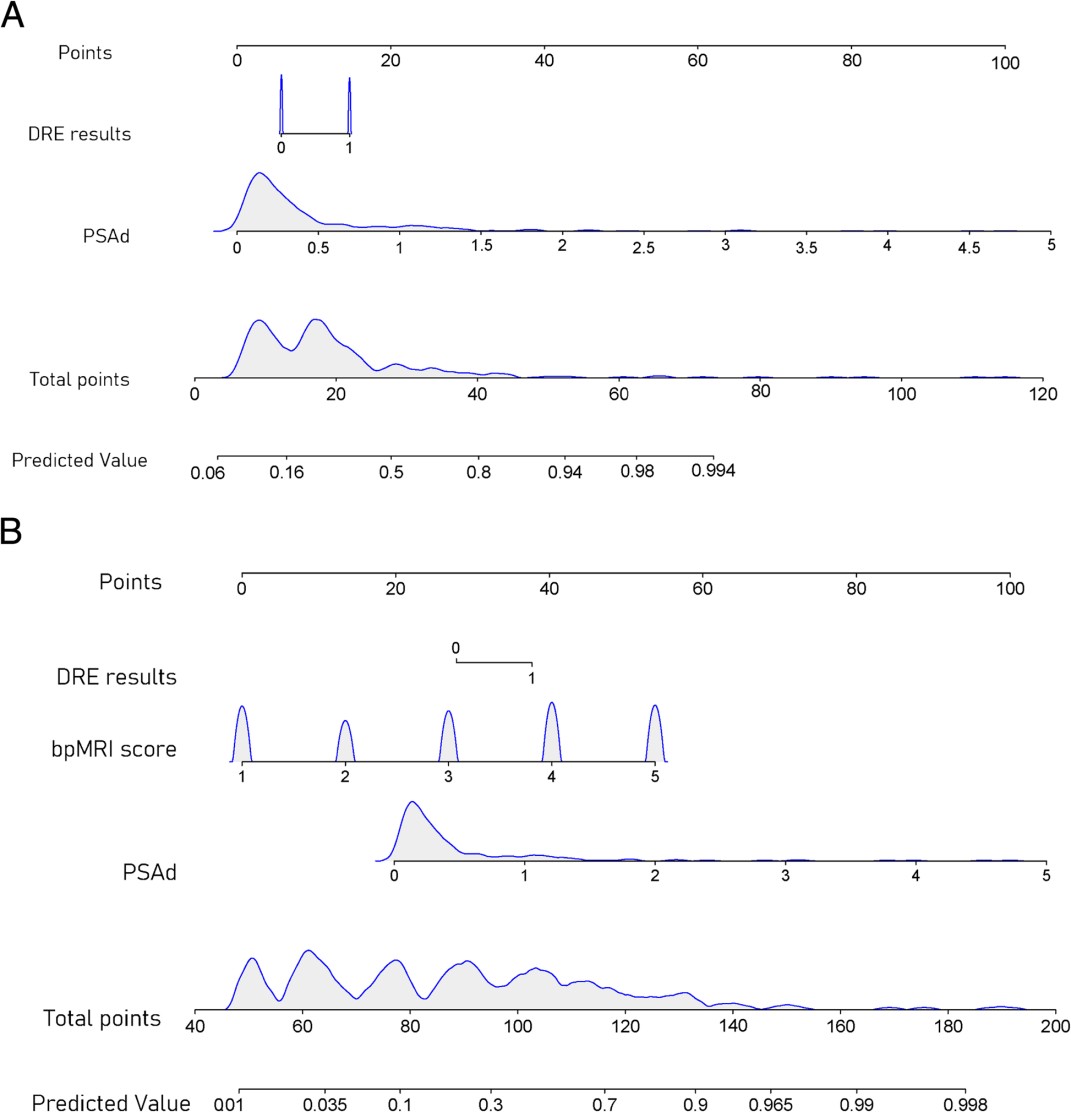
**A** Clinical nomogram predicting csPCa. The clinical nomogram predicting the probability of csPCa in patients undergoing prostate biopsy based on PSAd and DRE results. Instructions: locate the patient’s prebiopsy PSAd on the PSAd axis. Draw a line straight upward to the point axis to determine how many points toward the probability of csPCa the patient receives for his PSAd level. Repeat the process for each additional variable. Sum the points for each predictor. Locate the final sum on the total-point axis. Draw a line straight down to find the patient’s probability of having csPCa. **B** bpMRI PI-RADS v2.1-based nomogram predicting csPCa. The nomogram predicting the probability of csPCa in patients undergoing prostate biopsy based on the PSAd, DRE outcomes and the bpMRI PI-RADS v2.1 score. csPCa = Clinically significant prostate cancer; PSAd = prostate-specific antigen density; DRE = Digital rectal examination; PI-RADS v2.1, Prostate Imaging Reporting and Data System version 2.1; bpMRI = biparametric Magnetic Resonance Imaging

In multivariate analysis, PSAd (odds ratio [OR] = 13.22 [95% confidence interval (CI): 4.42–39.51], *p* < 0.001) and DRE outcomes (OR = 3.60 [95% CI: 1.58–8.24]), *p* = 0.002) were strongly associated with csPCa, and a clinical nomogram (clinical model) was constructed (Table 2; Fig. 2A). Multivariate logistic regression analysis showed that the PSAd (OR = 4.34 [95% CI: 1.38–13.69], *p* = 0.012), DRE results (OR = 2.73 [95% CI: 1.07–6.97], *p* = 0.036), and the bpMRI PI-RADS v2.1 score (OR = 2.96 [95% CI: 2.10–4.18], *p* < 0.001) were independent predictors for csPCa detection, and a bpMRI PI-RADS v2.1-based nomogram was developed (Fig. 2B).

**Table 2:** Logistic regression analysis for the detection of csPCa in the development cohort

	**Univariate**		**Multivariate**	
**Clinical model**	**OR (95% CI)**	***p* value**	**OR (95% CI)**	***p* value**
tPSA	1.04 (1.02–1.05)	< 0.001	1.00 (0.98–1.03)	0.806
fPSA	1.10 (1.04–1.17)	0.001	0.94 (0.85–1.05)	0.263
Age	1.02 (0.99–1.05)	0.132	1.03 (0.99–1.07)	0.119
Prostate volume	0.99 (0.99–1.00)	0.220		
PSAd	9.69 (4.50–20.86)	< 0.001	13.22 (4.42–39.51)	< 0.001
DRE findings	2.30 (1.37–3.88)	0.002	3.60 (1.58–8.24)	0.002
Family history	1.43 (0.85–2.40)	0.176	0.68 (0.30–1.55)	0.360
**bpMRI PI-RADS v2.1-based model**				
PI-RADS v2.1 score	3.66 (2.64–5.09)	< 0.001	2.96 (2.10–4.18)	< 0.001
tPSA	1.04 (1.02–1.05)	< 0.001	1.01 (0.98–1.04)	0.647
fPSA	1.10 (1.04–1.17)	0.001	0.93 (0.83–1.04)	0.213
Age	1.02 (0.99–1.05)	0.132	1.02 (0.98–1.07)	0.259
Prostate volume	0.99 (0.99–1.00)	0.220		
PSAd	9.69 (4.50–20.86)	< 0.001	4.34 (1.38–13.69)	0.012
DRE outcome	2.30 (1.37–3.88)	0.002	2.73 (1.07–6.97)	0.036
Family history	1.43 (0.85–2.40)	0.176	0.84 (0.32–2.19)	0.728

*PSA* prostate-specific antigen, *PSAd* prostate-specific antigen density, *DRE* digital rectal examination, *bpMRI* biparametric Magnetic Resonance Imaging, *PI-RADS v2.1* Prostate Imaging Reporting and Data System version 2.1, *OR* odds ratio, *CI* confidence interval

**Fig. 3:**
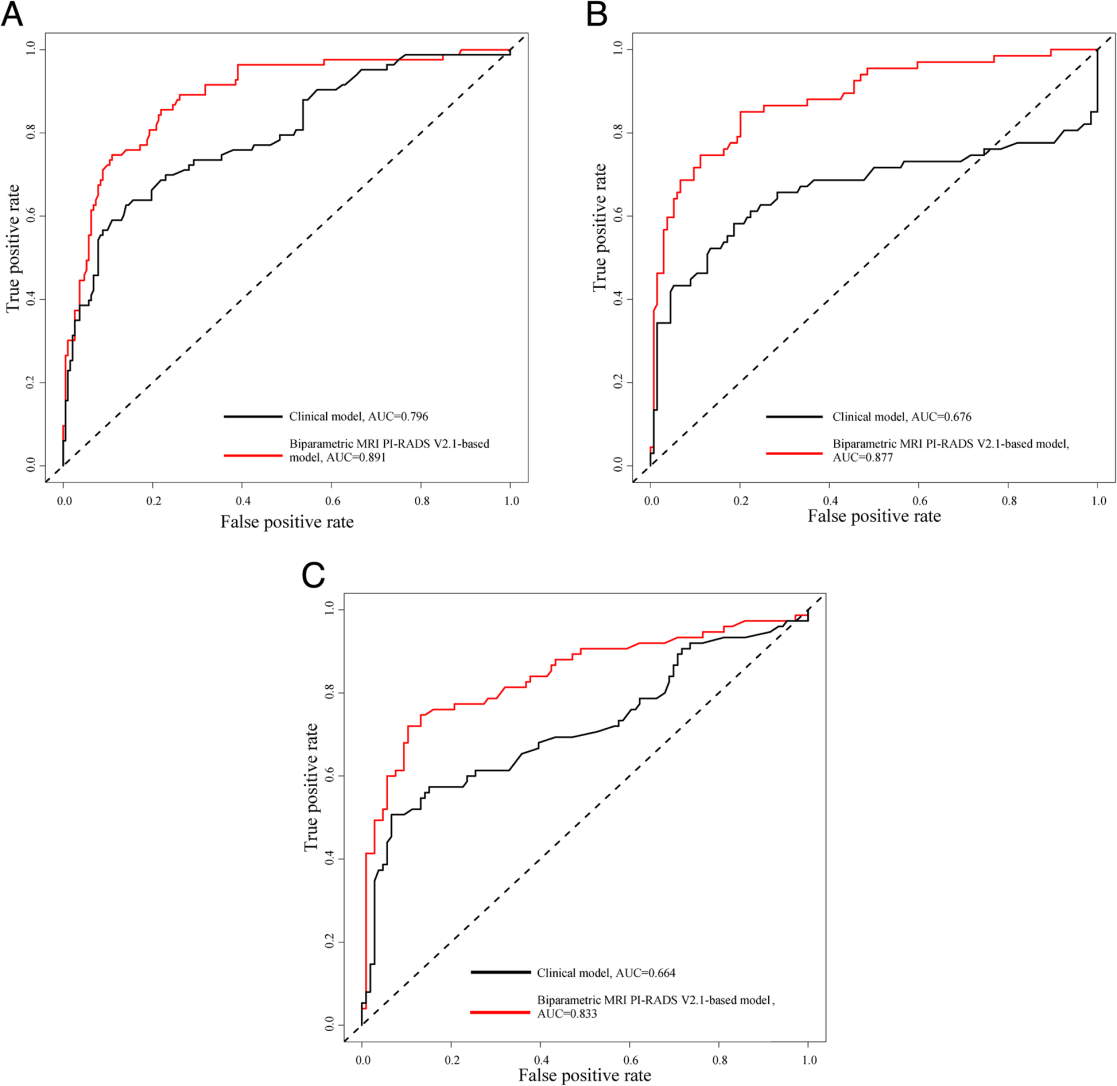
Receiver operating characteristic curves of risk prediction models for clinically significant prostate cancer in all cohorts. **A** Diagnosis of csPCa in the development cohort. **B** Diagnosis of csPCa in validation cohort 1. **C** Diagnosis of csPCa in validation cohort 2. csPCa = Clinically significant prostate cancer

**Fig. 4:**
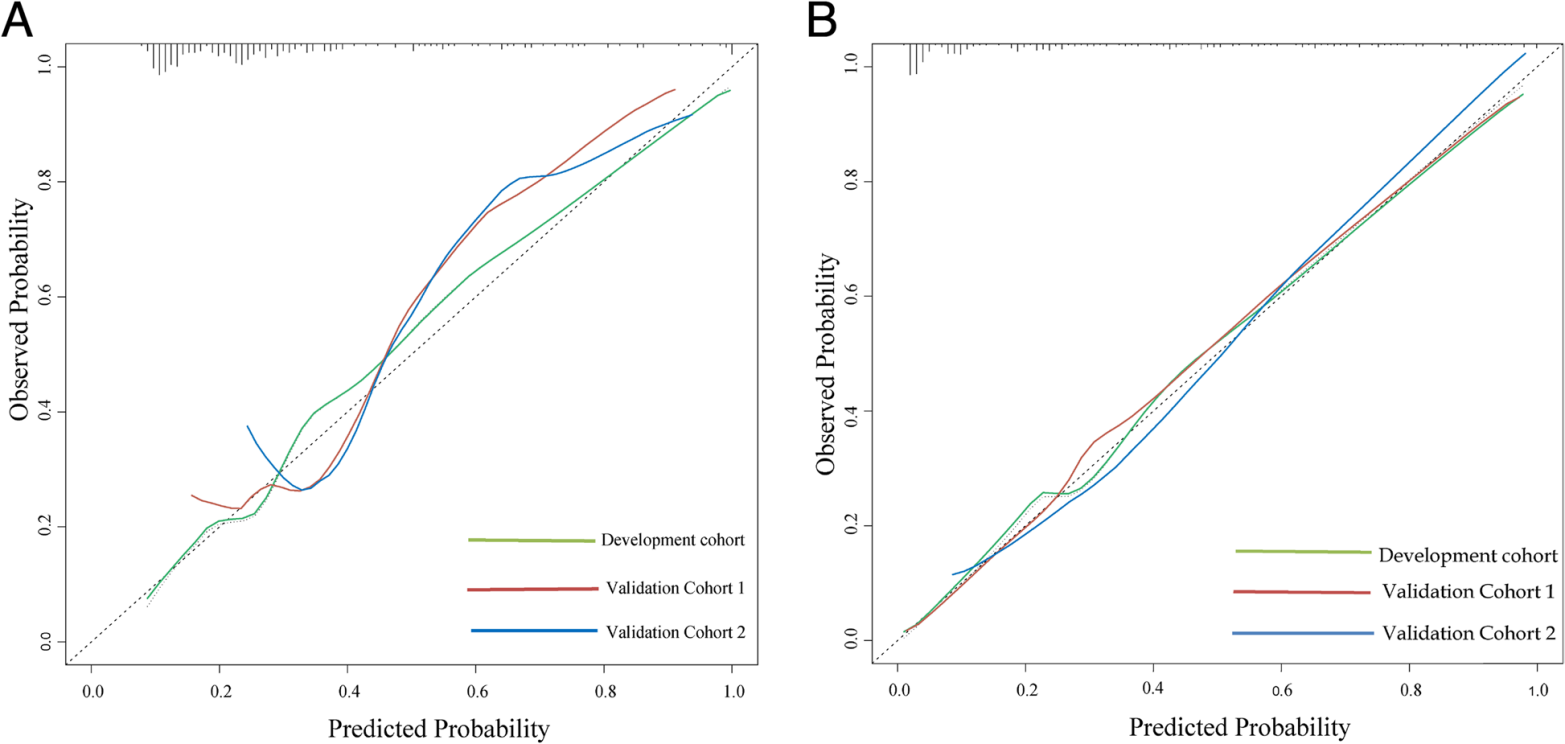
**A** Calibration curve of the clinical-based nomogram in the development and validation cohorts. **B** Calibration curve of the bpMRI PI-RADS v2.1-based nomogram in the development and validation cohorts. The calibration curves of the bpMRI PI-RADS v2.1-based model were similar to the standard curves in both cohorts. The curves were better than those in the calibration curve of the clinical model, indicating that bpMRI PI-RADS v2.1-based model had better predictive ability. PI-RADS v2.1 = Prostate Imaging Reporting and Data System version 2.1; bpMRI = biparametric Magnetic Resonance Imaging

ROC curve analysis showed that the bpMRI PI-RADS v2.1-based model significantly outperformed the clinical model in all cohorts (Table 3; Fig. 3). Using a fixed sensitivity for csPCa of 95% for the models, the specificity was higher for the bpMRI PI-RADS v2.1-based model than for the clinical model in all datasets (Table 3). In addition, the calibration curve of the bpMRI PI-RADS v2.1-based model were similar to the standard curves in all cohorts, which was better than the calibration curve of the clinical model, indicating that the bpMRI PI-RADS v2.1-based model has better predictive ability (Fig. 4).

**Table 3:** Performance comparison (AUC, 95% CI) of the different models and the DeLong test for significance

Cohorts	Clinical model			bpMRI PI-RADS v2.1-based model			*p* value for AUCs
	**Sensitivity%**	**Specificity%**	**AUC (95% CI)**	**Sensitivity%**	**Specificity%**	**AUC (95% CI)**	
**Development Cohort**	95	34	0.796(0.737, 0.856)	95	61	0.891 (0.849, 0.934)	< 0.001
**Validation Cohort 1**	95	0	0.676(0.586, 0.766)	95	51	0.877(0.826, 0.928)	< 0.001
**Validation Cohort 2**	95	8	0.664(0.577, 0.752)	95	19	0.833(0.768, 0.897)	< 0.001

*AUC* area under the curve, *CI* confidence interval, *bpMRI* biparametric Magnetic Resonance Imaging, *PI-RADS v2.1* Prostate Imaging Reporting and Data System version 2.1

#### Decision curve analysis and correlation analysis

**Fig. 5:**
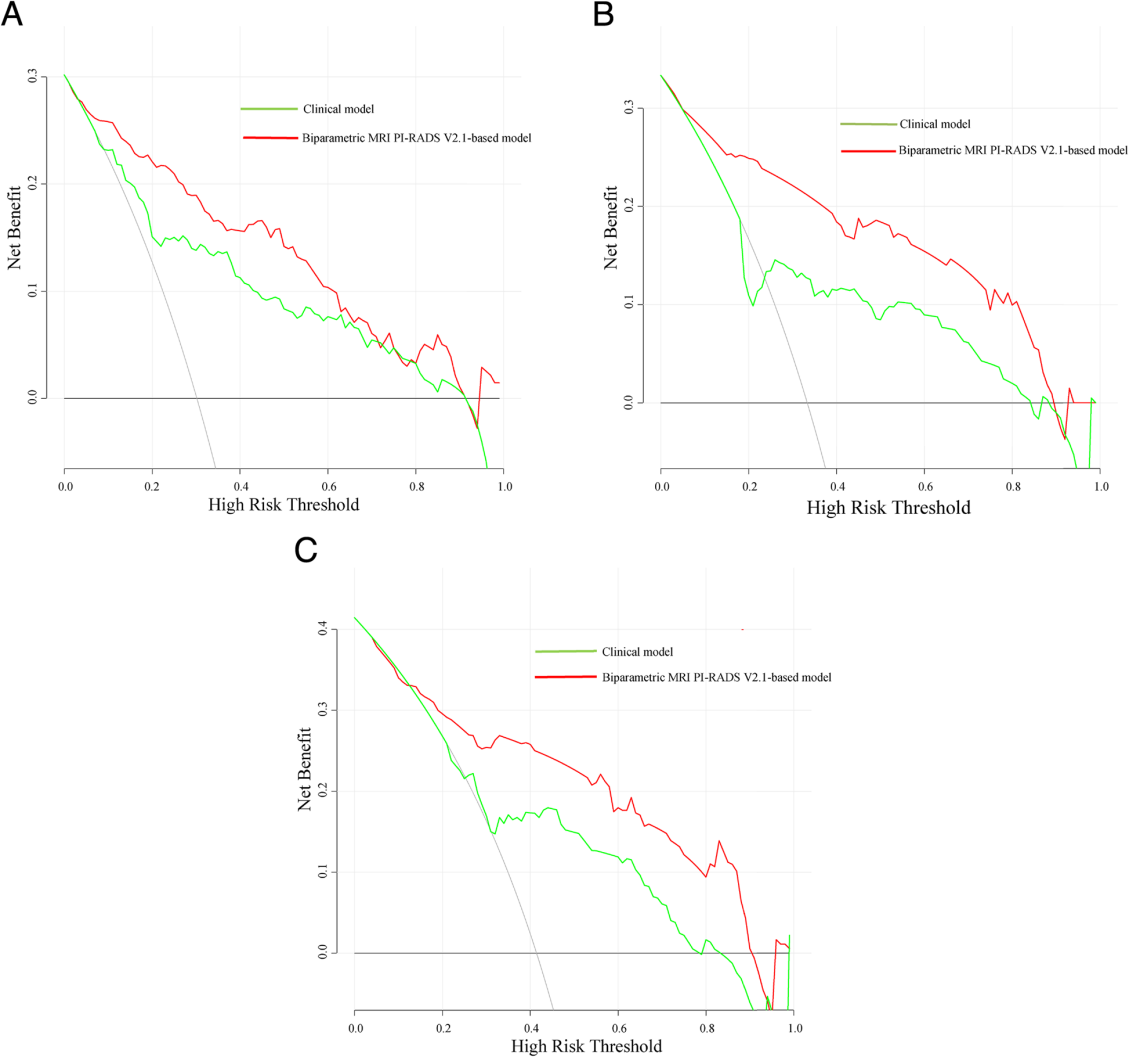
**A** Decision curve analysis (DCA) for the models for predicting csPCa in the development cohort. **B** DCA for the models for predicting csPCa in validation cohort 1. **C** DCA for the models for predicting csPCa in validation cohort 2. csPCa = Clinically significant prostate cancer; DCA = Decision curve analysis

**Fig. 6:**
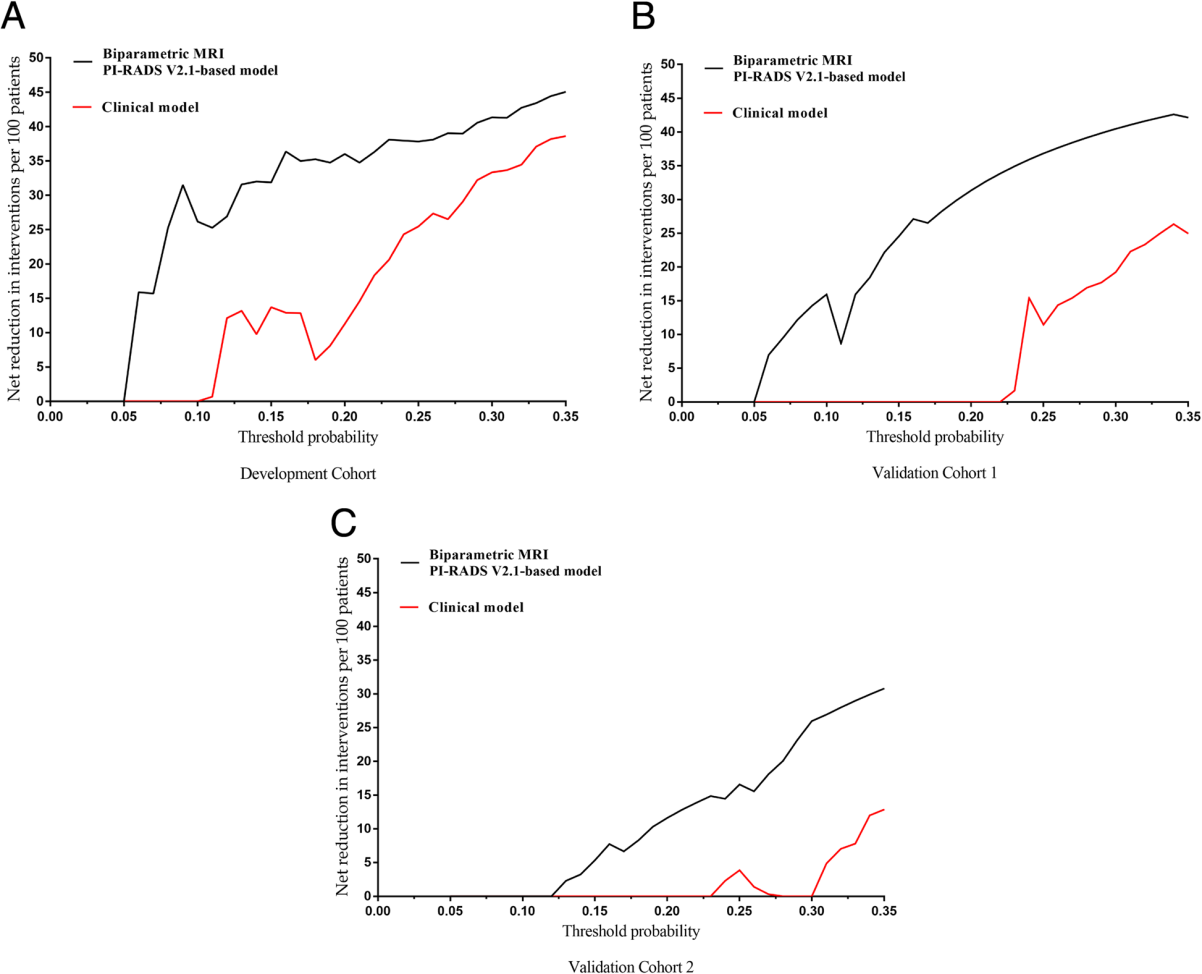
**A** Intervention avoidance curves in the development cohort. For example, with a probability threshold of 15%, the net reduction in interventions is approximately 14 per 100 patients by using the clinical model. In other words, at this probability threshold, the biopsies of patients on the basis of the clinical model are the equivalent of a strategy that reduced the biopsy rate by 14% without missing any cancers. The net reduction in interventions was approximately 32 per 100 patients by using the bpMRI PI-RADS v2.1-based model with a probability threshold of 15%. **B** Intervention avoidance curves in validation cohort 1. **C** Intervention avoidance curves in validation cohort 2. PI-RADS v2.1 = Prostate Imaging Reporting and Data System version 2.1; bpMRI = biparametric Magnetic Resonance Imaging

Figure 5 shows the net benefit in the development and validation cohorts. Figure 6 demonstrates the net reduction curves in all datasets. To assess the potential clinical benefit of both models, we performed a DCA using the different risk thresholds (10%-20%) in all cohorts. Overall, with increasing risk thresholds, more biopsies can be avoided (5–31 more patients per 100 patients can avoid biopsy) while maintaining a higher detection rate of csPCa (1–6.5 more csPCa patients per 100 patients can be detected) using the bpMRI PI-RADS v2.1-based model compared to the clinical model (Table 4). For example, compared to the clinical model, by applying the bpMRI PI-RADS v2.1-based model to validation cohort 1, a higher net benefit and net reduction in interventions per 100 patients was achieved at risk thresholds above 10%. For the biopsy strategy utilized in validation cohort 2, using the bpMRI PI-RADS v2.1-based model instead of the clinical model, the net benefit was higher at a risk threshold of 15%, with 1 additional csPCa patient detected per 100 patients (without a change in unnecessary biopsies).

**Table 4:** Biopsies avoided and test trade-off results for the constructed models to predict csPCa using different risk thresholds

Different risk thresholds	Clinical model			bpMRI PI-RADS v2.1-based model		
	**Overall biopsies avoided**	**Detected csPCa without unnecessary biopsies**	**Test trade-off, patients biopsied per detected csPCa**	**Overall biopsies avoided**	**Additional csPCa detected (without a change in unnecessary biopsies) when using the bpMRI PI-RADS v2.1-based model** **rather than the clinical model**	**Test trade-off, patients undergoing bpMRI per avoided unnecessary biopsy**
**Development Cohort**						
10%	0	23.2	4.3	26.1	2.6	3.8
15%	13.6	20.1	4.9	31.8	3.5	2.8
20%	11.2	15.1	6.6	36.0	6.5	1.5
**Validation Cohort 1**						
10%	0	25.9	3.8	15.9	1.8	5.5
15%	0	21.6	4.6	24.5	3.6	2.7
20%	0	10.9	9.1	31.3	14	0.7
**Validation Cohort 2**						
10%	0	34.9	2.8	0	0	0
15%	0	31.1	3.2	5.3	0.9	9.1
20%	0	26.8	3.7	11.6	2.8	3.5

*csPCa* clinically significant prostate cancer, *bpMRI* biparametric Magnetic Resonance Imaging, *PI-RADS v2.1* Prostate Imaging Reporting and Data System version 2.1

Because NB does not directly consider the cost and hazard of measuring predictors in the model, we used test trade-offs to compare the two models. In clinical practice, because bpMRI is a fast, noninvasive, and less costly testing protocol, the test trade-off can be considered low, so urologists would agree to perform 10 fewer bpMRIs to avoid one unnecessary biopsy in all cohorts with a risk threshold of 10–20% (Table 4).

Spearman correlation analysis showed that the prostate volume, tPSA, PSAd and bpMRI PI-RADS v2.1 score were significantly correlated with the ISUP grade (Table 5); bpMRI PI-RADS v2.1 scores were most strongly associated with PCa aggressiveness (correlation coefficient = 0.503, *p* < 0.001). Sensitivity analysis showed that eliminating missing values in the validation cohort had little effect on the net benefit of the bpMRI PI-RADS v2.1-based model, indicating that the main findings of this study are relatively robust (Table 6).

**Table 5:** Spearman correlation between clinical/MRI parameters and ISUP grade

	**Correlation coefficient (r)**	***p* value**
bpMRI PI-RADS v2.1 score	0.503	< 0.001
tPSA	0.143	< 0.001
fPSA	-0.004	0.901
Age	0.084	0.030
Prostate volume	-0.357	< 0.001
PSAd	0.279	< 0.001

*PSA* prostate-specific antigen, *PSAd* prostate-specific antigen density, *bpMRI* biparametric Magnetic Resonance Imaging, *PI-RADS v2.1* Prostate Imaging Reporting and Data System version 2.1, *ISUP* International Society of Urological Pathology

**Table 6:** Sensitivity analysis comparison of net benefits after excluding missing values in the validation cohorts

Risk thresholds	Validation Cohort 1	Validation Cohort 2
	**Net benefit (95% CI)**	**Net benefit (95% CI)**
**bpMRI PI-RADS v2.1-based model**		
10%	0.277 (0.210, 0.347)	0.340(0.268, 0.432)
15%	0.252 (0.191, 0.329)	0.320(0.242, 0.407)
20%	0.249 (0.178, 0.318)	0.296(0.222, 0.387)
**bpMRI PI-RADS v2.1-based model after excluding missing values**		
10%	0.308 (0.227, 0.386)	0.357 (0.276, 0.459)
15%	0.283 (0.210, 0.368)	0.338 (0.248, 0.427)
20%	0.271 (0.196, 0.348)	0.320 (0.228, 0.407)

*bpMRI* biparametric Magnetic Resonance Imaging, *PI-RADS v2.1* Prostate Imaging Reporting and Data System version 2.1, *CI* confidence interval

## Discussion

Biparametric MRI is increasingly being used to characterise prostate cancer. This study confirms that when bpMRI is incorporated into the prediction model, the model exhibits better fit and higher diagnostic accuracy, with fewer unnecessary biopsies compared to the clinical model. Furthermore, we demonstrated that the bpMRI PI-RADS v2.1 score was strongly correlated with the ISUP grade, which may give clinicians prebiopsy information about the aggressiveness of PCa. Finally, the results of sensitivity analysis demonstrated that our major findings were relatively reliable.

Remarkably, among the previously well-known risk calculators (ERSPC-RC and PCPT-RC), PSAd is not included. According to recent studies, PSAd not only predicted the outcome of the biopsy but was also a predictor of MRI equivocal lesions (PI-RADS score = 3) [24, 25]. In addition, Cuocolo et al. [26] demonstrated that PSAd derived from MRI correlated more significantly with PCa aggressiveness than the value measured from transrectal ultrasonography (TRUS). Therefore, it is not surprising that in our study, PSAd was one of the strong predictors in the clinical model. Similar to previously developed risk calculators [27, 28], our research also demonstrated that DRE findings are an independent predictor for csPCa detection. Overall, the clinical model achieved an AUC of 0.796 in detecting csPCa in the development cohort, which is consistent with previous studies [29, 30], suggesting that more efforts are needed to improve diagnostic accuracy.

In all cohorts of this study, the model based on the bpMRI-RADS v2.1 score performed better than the clinical model, as illustrated by the increase in AUC values. In addition, the clinical model was not calibrated as well as the bpMRI PI-RADS v2.1-based model. Importantly, with the increasing risk thresholds in DCA, the use of the bpMRI PI-RADS v2.1-based model can avoid more biopsies while maintaining higher csPCa detection rates compared to the clinical model, suggesting that the bpMRI PI-RADS v2.1-based model has higher value in reducing unnecessary biopsies. Understandably, the patient and the urologist can share the decision-making process to determine acceptable risk and biopsy thresholds to avoid missing csPCa [8]. In clinical practice, overtreatment of less invasive PCa decreases quality of life, but delayed treatment of csPCa increases treatment costs and mortality. Compared to mpMRI examination and biopsy, the bpMRI protocol is clearly a rapid, noninvasive, and less costly testing option, and the test trade-off can be considered low in clinical scenarios; therefore, the urologists would agree to perform 10 fewer bpMRIs to avoid one unnecessary biopsy with a risk threshold of 10–20%.

Novel risk tools based on clinical variables and additional genetic and/or protein-based biomarkers have been demonstrated to help avoid unnecessary biopsies, but they are laboratory dependent and expensive [27, 31]. In addition, in contrast to MRI-based stratification models, these risk models cannot determine the location or size of tumors within the prostate and therefore cannot be used to guide targeted biopsies. Multiple previous models that combine mpMRI findings with clinical variables have shown a 3–20% improvement in diagnostic accuracy [2, 18]. Recently, a small number of bpMRI-based nomograms have been developed [32–34]. In contrast to our study, a study by Boesen et al. [34] used bpMRI results to build a predictive nomogram. However, in his research, the bpMRI score was based on PI-RADS v2, and the results lacked external validation. The bpMRI-based nomograms constructed by Lee et al. [35] achieved a 92% diagnostic rate for csPCa, but nearly 80% of these patients underwent primary biopsies, which may have led to an overestimation of the diagnostic accuracy of the model. The implementation of prebiopsy mpMRI in all men with suspected PCa constitutes a fundamental paradigm shift in PCa diagnosis and could impose a tremendous financial and resource burden on the health care system [36]. Since the bpMRI protocol represents a cost-effective procedure, this also accounts for its lesion identification in terms of high sensitivity [37], which may be helpful in low-risk patients who might be candidates for active surveillance.

The ISUP grade is a measure of cancer aggressiveness. Several MRI-based functional parameters and radiomic signatures have been developed for the assessment of the biological aggressiveness of PCa [38–40]. However, no standardized imaging protocols have been available, and subjective measurements of the ROI depend on the experience and expertise of the radiologist, thus limiting the accuracy and reproducibility of the results. Previous studies concluded that the PI-RADS score can be used to predict lymph node involvement and extraprostatic extension [41] but only weakly correlates with the ISUP grade [42, 43]. In the present study, we demonstrated a strong correlation between the bpMRI PI-RADS v2.1 score and PCa aggressiveness, which is consistent with research by Pan et al. [32], suggesting that the bpMRI score can be used to predict the prebiopsy ISUP grade and potentially improve treatment planning.

Our study is not without limitations. First, this was a retrospective study, which may lead to patient selection bias. Further prospective, well-designed, large cohort studies are needed to confirm our findings. Second, the actual detection rate of csPCa may be underestimated compared to studies using template-mapped biopsies or whole-gland prostatectomy. Third, the data were interpreted by experienced radiologists at each institution using PI-RADS v2.1 for bpMRI; this may limit the generalizability of our results to less experienced institutions. Finally, assessing interobserver agreement for bpMRI PI-RADS v2.1 was beyond the scope of this study; however, van der Leest et al. [44] concluded that the interobserver agreement for biparametric MRI exceeded 90%.

## Conclusion

In conclusion, a predictive model based on the bpMRI score and clinical parameters is an easily accessible tool for avoiding unnecessary biopsies, and furthermore, bpMRI scores derived from PI-RADS v2.1 can be used to determine the aggressiveness of PCa prior to biopsy.

### Supplementary Information

**Additional file 1: Table S1. **bpMRI protocols.** Table S2.** Clinician experience-The following table details the experience of the clinicians involved in the present research.** Table S3. **Major packages of R software used in this study.

## Data Availability

The data that support the findings of this study are available from the corresponding author upon reasonable request.

## Abbreviations

AUC: Area under the curve
csPCa: Clinically significant prostate cancer
ERSPC: European Randomized Study of Screening for Prostate Cancer
PCPT-RC: Prostate Cancer Prevention Trial Risk Calculator
mpMRI: Multiparametric MRI
PCa: Prostate cancer
PI-RADS: Prostate Imaging Reporting and Data System
bpMRI: Biparametric MRI
DRE: Digital rectal examination
IQR: Interquartile range
MRI: Magnetic resonance imaging
PSA: Prostate-specific antigen
PSAd: Prostate specific antigen density
ISUP: International Society of Urological Pathology
CI: Confidence interval
