# Supplementary material for: Development and validation of a nomogram based on biparametric MRI PI-RADS v2.1 and clinical parameters to avoid unnecessary prostate biopsies
Source: BMC Med Imaging. 2023 Aug 15;23:106. doi: 10.1186/s12880-023-01074-7 (PMC10426075; doi:10.1186/s12880-023-01074-7)
Supplement: Supplementary file 1 — Additional file 1: Table S1. bpMRI protocols. Table S2. Clinician experience-The following table details the experience of the clinicians involved in the present research. Table S3. Major packages of R software used in this study. [file 12880_2023_1074_MOESM1_ESM.doc]

**Table S1. bpMRI protocols**

| **Site** | **Development Cohort** | **Validation Cohort 1** | **Validation Cohort 2** |
| --- | --- | --- | --- |
| **MRI manufacturer** | Siemens | Philips | Philips |
| **MRI Model** | Avanto | Ingenia | Achieva |
| **Field Strength** | 1.5T | 3T | 1.5T |
| **Coils** | PPA | PPA | PPA |
| **Sequences used** | T2, DWI | T2, DWI | T2, DWI |
| **T2 sequence details**  Planes acquired  Slice Thickness (axial)  Voxel size (axial) | Axial, coronal, sagittal  3mm  0.391 x 0.391 x 3.45 mm | Axial, coronal, sagittal  3mm  0.352 x 0.352 x 3 mm | Axial, coronal, sagittal  3mm  0.389 x 0.389 x 3 mm |
| **DWI sequence details**  B-values used  ADC threshold applied?  DWI combinations  Slice Thickness  Voxel size | 0,150,500, 1000,1400  No  Multi-b value, ADC & high b  5mm  1.51 x 1.51 x 5 mm | 0,800,1600  No  Multi-b value, ADC & high b  3mm  1.47 x 1.47 x 3mm | 0,100,800, 1600  No  Multi-b value, ADC & high b  3mm  0.893 x 0.893 x 3mm |
| **Bowel Relaxant** | Yes | Yes | Yes |

PPA, pelvic phased array; DWI, diffusion weighted imaging; ADC, Apparent Diffusion Coefficient; bpMRI, biparametric Magnetic Resonance Imaging

**Table S2. Clinician experience-The following table details the experience of the clinicians involved in the present research.**

| **Clinician category** | **Total number in study** | **Experience of individual clinicians prior to starting study** | |
| --- | --- | --- | --- |
|  |  | **Descriptor of experience** | **Median number, n (IQR)** |
| Prostate biopsy operators | 6 | Number of biopsies  performed per year  Number of years of experience | 94 (58-176)  7 (5.3-12.4) |
| Radiologists reporting MRI | 3 | Number of prostate MRIs  reported per year  Number of years of experience | 298 (223-421)  8.2 (6.3-15.2) |
| Pathologists reporting prostate specimens | 7 | Number of patient’s prostate  specimens analyzed/year  Number of years of experience | 130 (94-233)  7.2 (4.5-11.4) |

MRI, Magnetic Resonance Imaging; IQR, interquartile range

**Table S3. Major packages of R software used in this study.**

| **Functions** | **R package** |
| --- | --- |
| Logistic regression | autoReg |
| Building nomogram | rms |
| Calculation of C-index | rms |
| Decision curve analysis | rmda |

**Description of statistical quantities**

setwd(dir="C:/Users/niuxiangke/Desktop")

data<-read.csv("C:/Users/niuxiangke/Desktop/total prostate.csv")

library(gtsummary)

str(data)

library(tidyverse)

data <- data %>%

transmute(group,

Age = age,

tPSA = tPSA,

fPSA = fPSA,

volume = volume,

psad = psad,

gg = factor(data$gg),

pirads = factor(data$pirads),

outcome = factor(data$outcome),

DRE = factor(data$DRE),

history = factor(data$history))

str(data)

data %>% tbl_summary()

data%>% tbl_summary(by = group) %>% add_p()

**Fill in the missing values**

data<-read.csv("C:/Users/niuxiangke/Desktop/prostate1.1.csv")

library(lattice)

library(MASS)

library(nnet)

library(mice)

md.pattern(data)

imp <- mice(data, m = 4)

imp$imp$outcome

fit <- with(imp,lm(outcome ~ fPSA, data = data))

pooled <- pool(fit)

summary(pooled)

result <- complete(imp, action = 1)

result

options(max.print=10000)

**Regression model building**

setwd(dir="C:/Users/niuxiangke/Desktop")

data<-read.csv("C:/Users/niuxiangke/Desktop/prostate1.1.csv")

library(autoReg)

fit <- glm(outcome ~ tPSA + fPSA + age + volume + psad + DRE + history,

data=data,

family="binomial")

summary(fit)

autoReg(fit)

autoReg(fit, uni=TRUE, multi = TRUE, final=TRUE)

setwd(dir="C:/Users/niuxiangke/Desktop")

data<-read.csv("C:/Users/niuxiangke/Desktop/prostate1.1.csv")

library(autoReg)

fit <- glm(outcome ~ pirads + tPSA + fPSA + age + volume + psad + DRE + history,

data=data,

family="binomial")

summary(fit)

autoReg(fit)

autoReg(fit, uni=TRUE, multi = TRUE, final=TRUE)

**building nomogram**

library(rms)

data<-read.csv("C:/Users/niuxiangke/Desktop/prostate1.1.csv")

dd=datadist(data)

options(datadist="dd")

data$outcome<-factor(data$outcome, level=c(0,1))

fit<-lrm(outcome~pirads+psad + DRE,data=data)

nom1 <-nomogram(fit,fun=function(x)1/(1+exp(-x)),lp=TRUE,fun.at=c(0.0001,0.2,0.4,0.6,0.8,0.90), funlabel="Risk")

plot(nom1)

**Calculation of C-index for different models**

data<-read.csv("C:/Users/niuxiangke/Desktop/prostate1.1.csv")

formula<-as.formula(outcome~pirads+psad+DRE)

library(rms)

fit<-lrm(outcome~pirads+psad+DRE,data=data,x=T,y=T)

v<- validate(fit, method= "boot", B=1000, dxy=T)

Dxy = v[rownames(v)=="Dxy", colnames(v)=="index.corrected"]

orig_Dxy = v[rownames(v)=="Dxy", colnames(v)=="index.orig"]

bias_corrected_c_index <- abs(Dxy)/2+0.5

orig_c_index <- abs(orig_Dxy)/2+0.5

orig_c_index

bias_corrected_c_index

c<-rcorrcens(outcome~predict(fit),data=data)

lower<-c[1,1]-1.96*c[1,4]/2

upper<-c[1,1]+1.96*c[1,4]/2

cindex<-rbind(orig_c_index,lower,upper,bias_corrected_c_index)

cindex

data<-read.csv("C:/Users/niuxiangke/Desktop/prostate1.1.csv")

formula<-as.formula(outcome~psad+DRE)

library(rms)

fit<-lrm(outcome~pirads+psad+DRE,data=data,x=T,y=T)

v<- validate(fit, method= "boot", B=1000, dxy=T)

Dxy = v[rownames(v)=="Dxy", colnames(v)=="index.corrected"]

orig_Dxy = v[rownames(v)=="Dxy", colnames(v)=="index.orig"]

bias_corrected_c_index <- abs(Dxy)/2+0.5

orig_c_index <- abs(orig_Dxy)/2+0.5

orig_c_index

bias_corrected_c_index

c<-rcorrcens(outcome~predict(fit),data=data)

lower<-c[1,1]-1.96*c[1,4]/2

upper<-c[1,1]+1.96*c[1,4]/2

cindex<-rbind(orig_c_index,lower,upper,bias_corrected_c_index)

cindex

**Comparison of the area under the ROC curves of different models in the training set**

data<-read.csv("C:/Users/niuxiangke/Desktop/prostate1.1.csv")

data$outcome<-factor(data$outcome)

attach(data)

fit <- glm(outcome ~ pirads +psad + DRE,data=data,family = binomial())

pre <-predict(fit, data =data_data, type = "response")

fit1<- glm(outcome ~ DRE +psad,data=data,family = binomial())

Pre1 <-predict(fit1, data =data_data, type = "response")

data$prob<- predict(fit, newdata=data, type="response")

data$prob1<- predict(fit1, newdata=data, type="response")

detach(data)

library(pROC)

roc_pre1<-roc(data$outcome,Pre1)

roc_pre<- roc(data$outcome,pre)

roc.test(roc_pre1,roc_pre,method="delong")

**Comparison of the area under the ROC curves of different models in the validation group**

traindata<-read.csv("C:/Users/niuxiangke/Desktop/prostate1.1.csv")

testdata<-read.csv("C:/Users/niuxiangke/Desktop/prostate3.1.csv")

mylog<-glm (outcome ~ pirads + psad + DRE,family=binomial(link = "logit"),

data=traindata)

traindata$predict<-predict(newdata=traindata, mylog, "response" )

testdata$predict<-predict(newdata=testdata, mylog, "response" )

library(pROC)

trainroc<-roc (outcome~predict, data=traindata, smooth=F)

plot(trainroc, print.auc=TRUE, print.thres=TRUE, main="ROC curve in traindata",

identity.lty=1, identity.lwd=1)

auc(trainroc);ci(trainroc)

testroc<- roc (outcome~predict, data=testdata, smooth=F)

plot(testroc, print.auc=TRUE, print.thres=TRUE, main="ROC curve in testdata",

identity.lty=1, identity.lwd=1)

auc(testroc);ci(testroc)

mylog2<- glm (outcome ~ psad + DRE,family=binomial(link = "logit"),

data=traindata)

traindata$predict2<- predict(newdata=traindata, mylog2, "response" )

trainroc2<- roc (outcome~predict2,data=traindata, smooth=F)

plot(trainroc2, print.auc=TRUE, print.thres=TRUE, main="ROC curve in traindata",

identity.lty=1, identity.lwd=1)

roc.test(trainroc, trainroc2, method="delong")

mylog3<- glm (outcome ~ psad + DRE,family=binomial(link = "logit"),

data=testdata)

testdata$predict3<- predict(newdata=testdata, mylog3, "response" )

testroc3<- roc (outcome~predict3,data=testdata, smooth=F)

plot(testroc3, print.auc=TRUE, print.thres=TRUE, main="ROC curve in testdata",

identity.lty=1, identity.lwd=1)

roc.test(testroc, testroc3, method="delong")

**Calibration curves in development and validation cohort**

Pima.tr<-read.csv("C:/Users/niuxiangke/Desktop/prostate1.1.csv")

Pima.te<-read.csv("C:/Users/niuxiangke/Desktop/prostate2.1.csv")

Pima.ta<-read.csv("C:/Users/niuxiangke/Desktop/prostate3.1.csv")

str(Pima.tr)

str(Pima.te)

library(rms)

dd <- datadist(Pima.tr)

options(datadist='dd')

formula = as.formula(outcome ~ pirads + psad + DRE)

fit<-lrm(formula,data = Pima.tr, x=T,y=T)

nom<-nomogram(fit,

fun=function(x)1/(1+exp(-x)),

lp=F,

fun.at = c(0.05, 0.1,0.3,0.5,0.7,0.9),

funlabel = 'Risk')

plot(nom)

cal<-calibrate(fit, method = 'boot', B=1000, data = Pima.tr)

plot(cal,

xlim=c(0,1.0),ylim=c(0,1.0),

xlab = "Predicted Probability",

ylab = "Observed Probability"

)

pre <- predict(fit, newdata = Pima.te)

Pima.te = cbind(Pima.te, pre)

fit2 <- lrm(outcome ~ pre, data = Pima.te, x=T, y=T)

cal2<-calibrate(fit2, method = 'boot', B=1000, data = Pima.te)

plot(cal2,

xlim=c(0,1.0),ylim=c(0,1.0),

xlab = "Predicted Probability",

ylab = "Observed Probability"

)

pre <- predict(fit, newdata = Pima.ta)

Pima.ta = cbind(Pima.ta, pre)

fit3 <- lrm(outcome ~ pre, data = Pima.ta, x=T, y=T)

cal3<-calibrate(fit3, method = 'boot', B=1000, data = Pima.te)

plot(cal3,

xlim=c(0,1.0),ylim=c(0,1.0),

xlab = "Predicted Probability",

ylab = "Observed Probability")

plot(cal,

xlim=c(0,1.0),ylim=c(0,1.0),

xlab = "Predicted Probability",

ylab = "Observed Probability"

)

lines(cal[,c('predy','calibrated.corrected')],

# type="l",

lwd=2,lty=1,

col=c(rgb(60,179,113,maxColorValue = 255)),pch=14

) # bias-corrected

lines(cal2[,c('predy','calibrated.corrected')],

# type="l",

lwd=2,lty=1,

col=c(rgb(192,98,83,maxColorValue = 255)),pch=14

) # bias-corrected

lines(cal3[,c('predy','calibrated.corrected')],

# type="l",

lwd=2,lty=1,

col=c(rgb(0,118,192,maxColorValue = 255)),pch=14

) # bias-corrected

library(ResourceSelection)

model<-glm(formula,data=Pima.tr,family=binomial(link=logit))

hl<-hoslem.test(model$y,fitted(model),g=4)

hl

library(ResourceSelection)

model<-glm(formula,data=Pima.te,family=binomial(link=logit))

hl<-hoslem.test(model$y,fitted(model),g=4)

hl

library(ResourceSelection)

model<-glm(formula,data=Pima.ta,family=binomial(link=logit))

hl<-hoslem.test(model$y,fitted(model),g=4)

Hl

**Net benefit and net reduction curve in development cohort**

data<-read.csv("C:/Users/niuxiangke/Desktop/prostate1.1.csv")

formula1<-as.formula(outcome~pirads+psad+DRE)

formula2<-as.formula(outcome~psad+DRE)

library(rmda)

model1<-decision_curve(formula1,data=data,family = binomial(link='logit'),thresholds = seq(0,1,by=0.01),confidence.intervals = 0.95,study.design = 'cohort')

model2<-decision_curve(formula2,data=data,family = binomial(link='logit'),thresholds = seq(0,1,by=0.01),confidence.intervals = 0.95,study.design = 'cohort')

plot_clinical_impact(model1,population.size = 100,cost.benefit.axis = TRUE,

n.cost.benefit=8,col=c('red','blue'),

confidence.intervals=FALSE)

plot_clinical_impact(model2,population.size = 100,cost.benefit.axis = TRUE,

n.cost.benefit=8,col=c('red','blue'),

confidence.intervals=FALSE)

summary(model1,measure="NB")

summary(model2,measure="NB")

source("dca.R")

data.set<-read.csv("C:/Users/niuxiangke/Desktop/prostate1.1.csv")

attach(data.set)

#Test whether family history is associated with cancer

model = glm(outcome ~ DRE + psad, family=binomial(link="logit"), data=data.set)

data.set$predoutcome = predict(model, type="response")

dca(data=data.set, outcome="outcome", predictors="predoutcome", smooth="TRUE", probability="FALSE", intervention="TRUE",xstart=0.05, xstop=0.35)

**DCA curve**

data<-read.csv("C:/Users/niuxiangke/Desktop/prostate1.1.csv")

formula1<-as.formula(outcome~pirads+psad+DRE)

formula2<-as.formula(outcome~psad+DRE)

library(rmda)

model1<-decision_curve(formula1,data=data,family = binomial(link='logit'),thresholds = seq(0,1,by=0.01),confidence.intervals = 0.95,study.design = 'cohort')

model2<-decision_curve(formula2,data=data,family = binomial(link='logit'),thresholds = seq(0,1,by=0.01),confidence.intervals = 0.95,study.design = 'cohort')

plot_decision_curve(model1,curve.names=c('model1'), xlim=c(0,1),cost.benefit.axis =FALSE,col= c('red'), confidence.intervals=FALSE, standardize = FALSE)

plot_decision_curve(model2,curve.names=c('model2'), xlim=c(0,1),cost.benefit.axis =FALSE,col= c('red'), confidence.intervals=FALSE, standardize = FALSE)

model_all<-list(model1,model2)

plot_decision_curve(model_all,curve.names=c('model1','model2'), xlim=c(0,1),cost.benefit.axis =FALSE,col= c('red','green'), confidence.intervals=FALSE, standardize = FALSE, legend.position = "none")

**Correlation analysis**

setwd(dir="C:/Users/niuxiangke/Desktop")

data<-read.csv("C:/Users/niuxiangke/Desktop/total prostate.csv")

cor.test( ~ psad + gg,

data=data,

method = "spearman",

continuity = FALSE,

conf.level = 0.95)
